# Association Between Dietary Regimen and Renal Function Parameters in African Pygmy Hedgehogs (*Atelerix albiventris*)

**DOI:** 10.3390/ani16132066

**Published:** 2026-07-04

**Authors:** Kristina Spariosu, Ana Pešić, Ksenija Nešić, Diana Brozić, Jelena Francuski Andrić, Branislav Vejnović, Miloš Vučićević

**Affiliations:** 1 Department of Pathophysiology, Faculty of Veterinary Medicine, University of Belgrade, 11000 Belgrade, Serbia; kristina@vet.bg.ac.rs (K.S.); jelenaf@vet.bg.ac.rs (J.F.A.); 2 Department of Equine, Small Animal, Poultry and Wild Animal Diseases, Faculty of Veterinary Medicine, University of Belgrade, 11000 Belgrade, Serbia; ana.pesic@vet.bg.ac.rs; 3 Institute of Veterinary Medicine of Serbia, 11000 Belgrade, Serbia; ksenija.nesic@gmail.com; 4 Center for Animal Nutrition and Welfare, University of Veterinary Medicine Vienna, 1210 Vienna, Austria; diana.brozic@vetmeduni.ac.at; 5 Department of Economics and Statistics, Faculty of Veterinary Medicine, University of Belgrade, 11000 Belgrade, Serbia; branislavv@vet.bg.ac.rs

**Keywords:** African pygmy hedgehog, *Atelerix albiventris*, chronic kidney disease, nutrition, dietary regimen, renal biomarkers, phosphorus, exotic pet medicine, serum biochemistry, hedgehog diet

## Abstract

African pygmy hedgehogs (APHs) are increasingly kept as companion animals, yet evidence-based nutritional recommendations for this species remain limited. Commercial cat or kitten diets are still commonly used by owners and veterinarians in practice, despite being formulated exclusively for domestic cats rather than for hedgehogs with omnivorous–insectivorous feeding habits. This cross-sectional study evaluated the association between dietary regimen and serum biochemical parameters associated with renal function in APHs. Blood samples were collected from 19 client-owned APHs aged 12–68 months: 10 hedgehogs fed a commercial kitten diet and 9 fed a commercial APH-specific diet. Serum concentrations of blood urea nitrogen, creatinine, phosphorus, and calcium were measured, and the nutritional composition of the two diets was compared using manufacturer declarations and targeted laboratory analyses. Hedgehogs fed the commercial kitten diet had significantly higher serum blood urea nitrogen (*p* = 0.0133), creatinine (*p* = 0.0279), and phosphorus (*p* = 0.0279) concentrations than those fed the APH-specific diet, whereas serum calcium concentrations did not differ significantly (*p* = 0.3846). These differences occurred despite similar dietary phosphorus content, while the commercial kitten diet had higher declared fat and lower fiber content. The findings suggest that dietary regimen may be associated with alterations in renal biochemical profiles in APHs and support the use of species-appropriate diets in clinical practice.

## Simple Summary

African pygmy hedgehogs are increasingly kept as pets, but their optimal diet is still not well defined. In practice, many owners feed them commercial cat or kitten food, although these diets are formulated for cats rather than hedgehogs. This study compared serum biochemical parameters associated with kidney function in hedgehogs fed a commercial kitten diet and those fed an African pygmy hedgehog-specific diet. Hedgehogs fed a kitten diet had higher blood urea nitrogen, creatinine, and phosphorus concentrations, while calcium concentrations did not differ between groups. These findings suggest that long-term feeding of non-species-specific diets may be associated with less favorable renal biochemical profiles in African pygmy hedgehogs. Further studies are needed, but the results support the use of diets formulated specifically for this species.

## 1. Introduction

African pygmy hedgehogs (APHs), also known as four-toed hedgehogs (*Atelerix albiventris*), have become increasingly popular as exotic companion pets and are occasionally used as animal models in biomedical and toxicological research [1]. Their unique gastrointestinal anatomy, characterized by the absence of a caecum, together with species-specific metabolic requirements, underscores the importance of appropriate nutritional management for maintaining health [2]. In the wild, APHs are opportunistic feeders, consuming a diverse diet composed primarily of invertebrates, supplemented with small vertebrates and plant material [3,4].

Under captive conditions, recommended feeding practices typically include commercially formulated APH-specific diets combined with insects, limited amounts of fruits and vegetables, and occasional cooked animal protein [5,6]. Despite these recommendations, commercial kitten diet remains widely used in clinical practice due to availability, convenience, and owner familiarity [7]. This practice persists even though feline diets are formulated for obligate carnivores and may differ substantially from the nutritional requirements of these omnivorous mammals with predominantly insectivorous dietary habits, raising concerns regarding long-term metabolic and organ-specific effects.

Chronic kidney disease (CKD) is commonly reported in APHs, particularly in older and geriatric individuals, typically those over 3 years of age, given the average lifespan of 4–6 years in captive animals [8,9]. Clinical manifestations such as polyuria, polydipsia, weight loss, and lethargy are often observed only in advanced stages of disease, limiting opportunities for early intervention [10]. Unlike in dogs and cats, validated staging systems for CKD, species-specific diagnostic thresholds, and standardized interpretation of renal biomarkers such as blood urea nitrogen (BUN), creatinine (CREA), phosphorus (PHOS), symmetric dimethylarginine (SDMA), and cystatin C (CysC) have not been established for APHs. Age and suspected genetic predisposition are recognized contributors to renal pathology; however, potentially modifiable factors, including long-term dietary regimen, have received limited attention.

The existing literature on renal disease in APHs primarily consists of *post-mortem* pathological descriptions and retrospective clinical observations, without systematic evaluation of nutritional influences on renal physiology [8,11]. To date, no studies have specifically assessed the relationship between dietary regimens and serum renal function parameters in this species. Moreover, nutritional recommendations for APHs are frequently extrapolated from other taxa, highlighting a significant gap in evidence-based dietary guidance [4,5].

Therefore, the aim of the present study was to evaluate the association between dietary regimen and serum renal function parameters in APHs, specifically comparing commercial kitten diet with a commercial APH-specific diet. By examining biochemical indicators of renal function in relation to diet, this study seeks to provide clinically relevant data to support species-appropriate nutritional practices in captive APHs.

## 2. Materials and Methods

### 2.1. Study Design and Animals

This cross-sectional observational study included 19 privately owned pet APHs, comprising 9 males and 10 females from different households in Serbia. The study involved client-owned hedgehogs presented for clinical evaluation and did not include any dietary intervention or longitudinal follow-up. The animals’ ages ranged from 12 to 68 months. All hedgehogs were presented for clinical evaluation at the Small Animal Teaching Hospital, Faculty of Veterinary Medicine, University of Belgrade, Serbia. When present, reported clinical signs were non-specific (e.g., reduced appetite and lethargy), and diet grouping was performed independently of the presenting complaint. No animal was allocated to a dietary group on the basis of clinical diagnosis or the severity of clinical signs.

Dietary history was obtained from the owners prior to clinical examination. According to owner reports, all included hedgehogs had been fed their respective diets since being acquired by their owners, without major dietary changes prior to presentation. Based on the predominant diet fed, animals were allocated into two groups: those fed a commercial kitten diet (“Kitten diet” group) and those fed a commercial APH-specific diet (“APH-specific diet” group). Ten hedgehogs (5 males, 5 females) were fed a commercial kitten diet, while nine hedgehogs (4 males, 5 females) received an APH-specific diet. Selected analytical components of both diets are presented in Table 1.

Only hedgehogs with a consistent owner-reported long-term dietary regimen were included in the study. Animals receiving frequent changes or mixed feeding practices without a clearly predominant diet were excluded from the analyses. The exact duration of dietary exposure varied among individuals and was based on owner reports but could not be objectively verified.

Dietary composition was assessed based on available manufacturer declarations, supplemented by targeted laboratory analysis when specific parameters were not provided. Based on the nutritional composition, the calculated calcium-to-phosphorus ratios were 1.25 for the commercial kitten diet and 2.30 for the APH-specific diet.

### 2.2. Clinical and Ultrasonographic Examination

All animals underwent a complete clinical examination followed by abdominal ultrasonography using a Vetus 8 ultrasound system (Shenzhen Mindray Bio-Medical Electronics Co., Ltd., Shenzhen, China). Ultrasonographic examination was performed as part of the clinical evaluation of renal status, as ultrasonography is considered a valuable adjunct diagnostic tool in the assessment of CKD in companion animals [13].

As reference values for renal size, corticomedullary differentiation, renal parenchymal echogenicity, and Doppler hemodynamic parameters have not yet been established for APHs, renal status was assessed qualitatively. Ultrasonographic findings considered suggestive of renal pathology included subjectively altered renal parenchymal echogenicity, reduced or absent corticomedullary differentiation [14], irregular renal contours [15], the presence of renal cysts [16], and qualitative assessment of kidney size and contours as part of the overall renal evaluation [17]. All included hedgehogs exhibited one or more ultrasonographic findings considered suggestive of renal alterations; however, biochemical abnormalities varied in magnitude among individuals. Renal size was not used as a discriminative parameter, as kidneys did not show consistent size alterations. Instead, qualitative structural ultrasonographic changes were considered the primary indicators of renal involvement.

### 2.3. Laboratory Analysis

Blood samples were collected once from each hedgehog during the clinical evaluation, and no repeated or follow-up sampling was performed. Owners were instructed to withhold food for at least 8 h prior to presentation in order to minimize potential postprandial variation in serum biochemical parameters. However, compliance with these instructions could not be objectively verified.

Following inhalation anesthesia with sevoflurane (2%; Sevoflurane Baxter, Baxter S.A., Lessines, Belgium), as previously described for APHs [18,19], blood samples were collected from *vena cava cranialis*. No gross hemolysis was observed in any of the serum samples. Serum concentrations of renal function parameters, including BUN, CREA, PHOS, and calcium, were determined using an automated biochemical analyzer (Mindray BS-240, Shenzhen Mindray Bio-Medical Electronics Co., Ltd., Shenzhen, China) and corresponding commercial reagents.

Urine samples were obtained from only two animals; due to the limited sample size, urinalysis results were not included in the present study, and CKD staging based on urinary parameters could not be performed.

### 2.4. Ethical Statement

Written informed consent was obtained from all owners prior to sample collection and inclusion of animals in the study. The study was conducted in accordance with the approval issued by the Ministry of Agriculture, Forestry and Water Management of the Republic of Serbia (Approval No. 000896071 2023 14841 002 000 323 022, 8 March 2024), based on the opinion of the Ethics Committee of the Faculty of Veterinary Medicine, University of Belgrade.

### 2.5. Statistical Analysis

Data distribution for age and serum renal function parameters (BUN, CREA, PHOS, and calcium) was assessed using the Shapiro–Wilk test for normality. As normality was rejected for all analyzed variables (*p* < 0.05), data are presented as medians with interquartile ranges (IQR). Differences between dietary groups were evaluated using the Mann–Whitney U test. Correlations between serum renal function parameters were assessed using Spearman’s rank correlation coefficient. Statistical significance was set at *p* < 0.05. All statistical analyses were performed using GraphPad Prism software, version 7 (GraphPad Software Inc., San Diego, CA, USA).

## 3. Results

**Figure 1:**
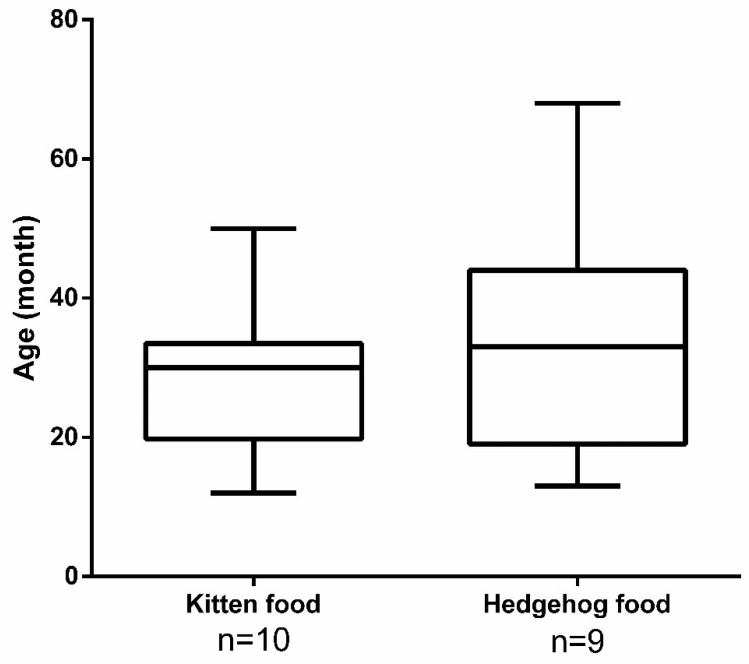
Age distribution of African pygmy hedgehogs fed commercial kitten diet or an APH-specific diet. Boxplots represent the interquartile range (box), median value (horizontal line), and minimum–maximum range (whiskers).

No statistically significant difference in hedgehog age was observed between the Kitten diet group (*n* = 10) and the APH-specific diet group (*n* = 9). The median age was 30.00 months (IQR: 19.75–33.50) in the Kitten diet group and 33.00 months (IQR: 19.00–44.00) in the APH-specific diet group (Figure 1). The two dietary groups were also comparable with respect to sex distribution (Kitten diet: 5 males, 5 females; APH-specific diet: 4 males, 5 females).

Clinical presentation, physical examination findings, and renal ultrasonographic abnormalities are summarized in Table 2. The majority of hedgehogs were presented with non-specific clinical signs. Most animals had normal hydration status and maintained body condition at presentation, whereas dehydration, weight loss, or muscle atrophy were observed only in a minority of animals (Table 2). Cardiovascular examination did not reveal clinically relevant abnormalities in any of the examined hedgehogs.

**Figure 2:**
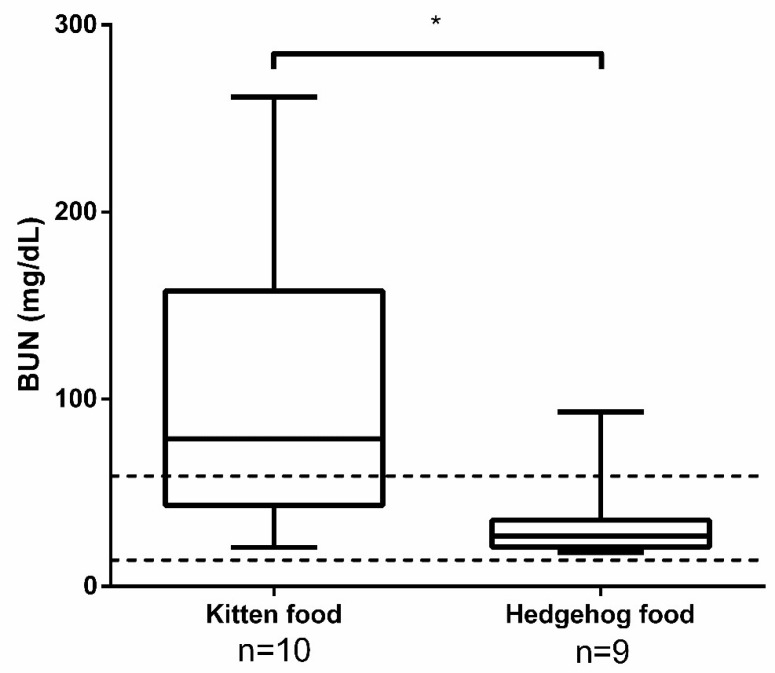
Serum blood urea nitrogen (BUN) concentrations in African pygmy hedgehogs fed commercial kitten diet or an APH-specific diet. Boxplots represent the interquartile range (box), median value (horizontal line), and minimum–maximum range (whiskers). Dashed lines indicate published reference intervals for African pygmy hedgehogs in the literature. * *p* < 0.05.

Significant differences between dietary groups were detected for all evaluated renal function parameters (Mann–Whitney U test: BUN, *p* = 0.0133; CREA, *p* = 0.0279; PHOS, *p* = 0.0279). Serum BUN concentrations were markedly higher in hedgehogs fed commercial kitten diet, with a median value of 78.83 mg/dL (IQR: 43.32–157.90), compared with 26.88 mg/dL (IQR: 21.22–35.39) in animals fed the APH-specific diet (median difference: +51.95 mg/dL; Figure 2). Although all animals were evaluated for suspected renal pathology, the magnitude of biochemical alterations differed significantly between dietary groups.

**Figure 3:**
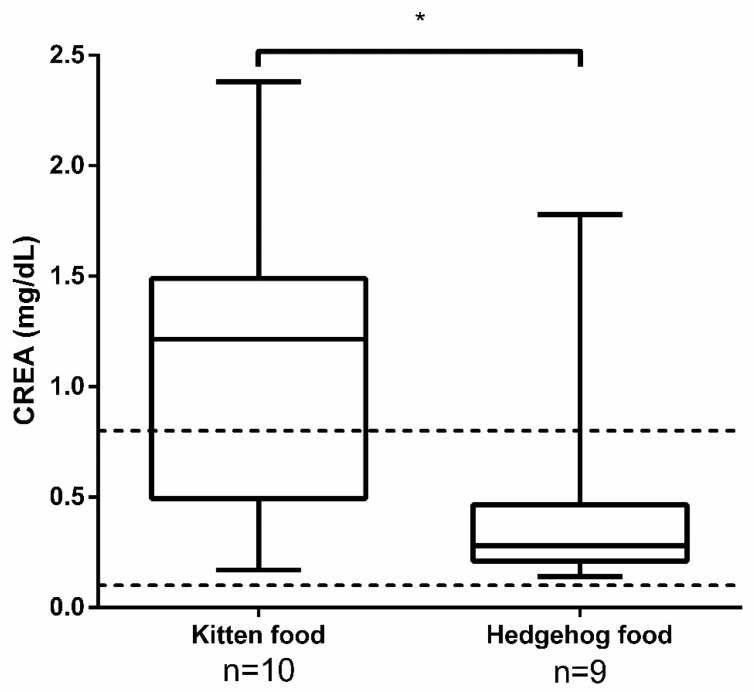
Serum creatinine (CREA) concentrations in African pygmy hedgehogs fed commercial kitten diet or an APH-specific diet. Boxplots represent the interquartile range (box), median value (horizontal line), and minimum–maximum range (whiskers). Dashed lines indicate published reference values reported for African pygmy hedgehogs in the literature. * *p* < 0.05.

Similarly, serum CREA concentrations were significantly elevated in the Kitten diet group, with a median value of 1.22 mg/dL (IQR: 0.49–1.49), compared with 0.28 mg/dL (IQR: 0.21–0.47) in the APH-specific diet group (median difference: +0.94 mg/dL; Figure 3). Creatinine values exceeding ranges commonly reported for APHs in the literature were observed in the majority of hedgehogs fed the commercial kitten diet [18,19].

**Figure 4:**
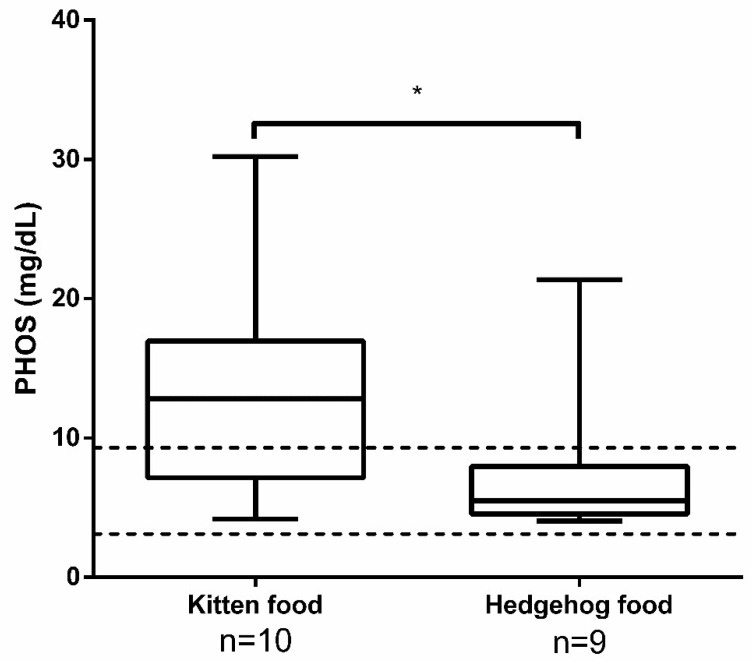
Serum phosphorus (PHOS) concentrations in African pygmy hedgehogs fed commercial kitten diet or an APH-specific diet. Boxplots represent the interquartile range (box), median value (horizontal line), and minimum–maximum range (whiskers). Dashed lines indicate published reference values reported for African pygmy hedgehogs in the literature. * *p* < 0.05.

Serum PHOS concentrations were also significantly higher in the Kitten diet group, with a median value of 12.81 mg/dL (IQR: 7.14–16.95), compared with 5.48 mg/dL (IQR: 4.54–7.96) in the APH-specific diet group (median difference: +7.33 mg/dL; Figure 4).

**Figure 5:**
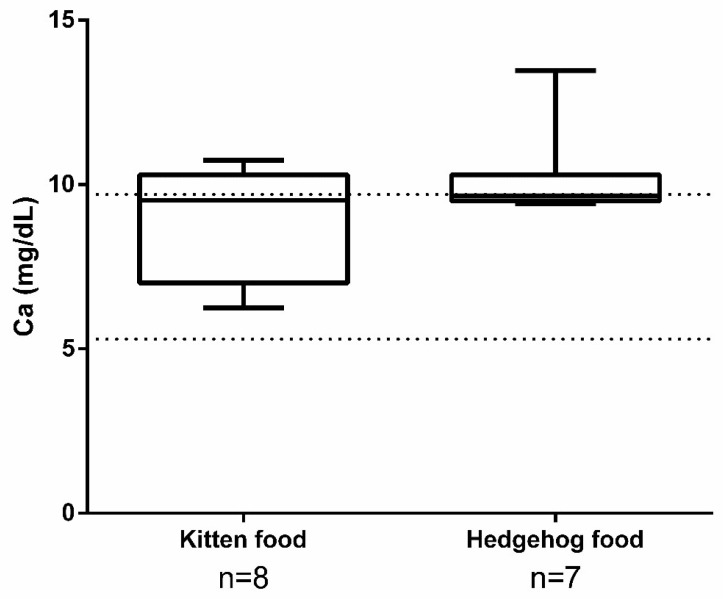
Serum calcium (Ca) concentrations in African pygmy hedgehogs fed commercial kitten diet or an APH-specific diet. Boxplots represent the interquartile range (box), median value (horizontal line), and minimum–maximum range (whiskers). Dashed lines indicate published reference values reported for African pygmy hedgehogs in the literature.

Serum calcium concentrations did not differ significantly between dietary groups. Median serum calcium concentration was 9.52 mg/dL (IQR: 7.71–9.90) in hedgehogs fed the commercial kitten diet and 9.66 mg/dL (IQR: 9.52–10.06) in animals fed the APH-specific diet (median difference: −0.14 mg/dL; Mann–Whitney U test, *p* = 0.3846; Figure 5).

A strong positive correlation was observed between serum CREA and PHOS concentrations across all examined hedgehogs (Spearman’s r = 0.718, *p* = 0.0005), indicating that higher PHOS concentrations were associated with higher CREA concentrations.

## 4. Discussion

The present study evaluated the association between different dietary regimens and renal function parameters in APHs of comparable age. Hedgehogs fed commercial kitten diet exhibited significantly higher serum concentrations of BUN, CREA, and PHOS compared with animals fed an APH-specific diet. In contrast, biochemical values in the APH-specific diet group remained within ranges commonly reported for apparently healthy APHs in the literature.

Renal pathology has been frequently reported in APHs, with necropsy-based studies reporting renal alterations in up to 50% of examined animals [11]. However, the clinical relevance of such findings remains unclear, as some lesions may represent incidental or subclinical changes. Reported prevalence of renal disease in APHs ranges from 1% to 9.43% [8,20], although these studies did not include information on dietary regimens. Consequently, the contribution of nutrition to renal health in this species has remained largely unexplored.

Age is a well-recognized risk factor for the development of CKD in humans, dogs, and cats [21,22,23]. African pygmy hedgehogs have a relatively short lifespan, typically 4 to 6 years, with individuals older than 3 years considered geriatric [9]. In the present study, the median age of animals in both dietary groups exceeded 30 months, indicating that age-related renal changes may have contributed to the observed biochemical alterations. Potential prerenal contributors to azotemia and hyperphosphatemia, such as dehydration or reduced water intake, were considered. However, no owner-reported decrease in water consumption was noted although owner-reported water intake is inherently subjective, and clinical examination did not reveal overt signs of dehydration at presentation. While transient prerenal influences cannot be entirely excluded, the consistent elevation of renal parameters across individuals within the same dietary group suggests that prerenal factors alone are unlikely to account for the observed differences. However, as age distribution did not differ significantly between groups, diet appears to represent an additional factor associated with altered renal parameters.

Assessment of renal dysfunction in APHs is challenging due to the absence of species-specific diagnostic criteria and staging systems. Interpretation of renal ultrasonographic findings in APHs remains challenging due to the absence of validated species-specific reference values for renal size, parenchymal echogenicity, and Doppler parameters. Nevertheless, ultrasonography still represents an important adjunct tool in the assessment of renal disease in companion animals and was therefore incorporated into the present evaluation [24,25]. Similarly, early markers of kidney damage, such as SDMA and CysC, widely used in dogs and cats, have not yet been validated in APHs. Therefore, the present study relied on ultrasonographic findings in combination with established late markers of kidney damage (BUN, CREA, and PHOS), representing currently available diagnostic tools for this species. In the present study, renal involvement was primarily identified based on qualitative ultrasonographic alterations rather than kidney size, as structural changes were consistently observed even in the absence of marked size differences.

Dietary composition may influence renal function through multiple mechanisms. High dietary protein intake has been associated with glomerular hyperfiltration and increased intraglomerular pressure in humans, potentially accelerating renal damage over time [26,27,28]. Nevertheless, in obligate carnivorous species, such as cats with intact renal function, clear evidence supporting this relationship remains limited. Although both diets, based on manufacturer declarations, contained similar crude protein levels within the recommended range for APHs [4], CREA concentrations were significantly higher in the kitten diet group. This finding suggests that protein source, amino acid profile, or processing conditions of feline diets may differ from species-specific nutritional requirements of APHs, emphasizing the importance of species-appropriate protein sources. However, as amino acid composition and protein digestibility were not evaluated in the present study, these proposed mechanisms remain speculative.

Phosphorus homeostasis is tightly regulated by intestinal absorption and renal excretion, mediated by endocrine mechanisms involving fibroblast growth factor 23 and parathyroid hormone [29,30]. In healthy individuals, excess dietary PHOS is efficiently excreted by the kidneys, maintaining serum concentrations within physiological limits. However, impaired renal excretory capacity can lead to hyperphosphatemia even in the absence of excessive dietary intake [31]. In the present study, serum PHOS concentrations were significantly higher in hedgehogs fed a commercial kitten diet, despite similar PHOS content reported for the two diets based on manufacturer declarations and targeted spectrometric analysis of the APH-specific diet [12]. These findings suggest that elevated serum PHOS may have been associated with reduced renal excretory capacity rather than increased dietary PHOS intake, potentially reflecting renal dysfunction in this group. Although serum PHOS concentrations may be artificially increased by sample hemolysis, no gross hemolysis was observed in any of the analyzed serum samples, making hemolysis an unlikely explanation for the observed differences. Another possible explanation could lie in differences in the content of inorganic PHOS salts between dietary regimens. Namely, inorganic PHOS salts are added to diets for various technical (e.g., water-binding, preservation, and texture modification) and nutritional purposes. In cats, soluble inorganic PHOS salts have been shown to exert a greater impact on PHOS homeostasis and renal health than organically bound PHOS [32,33]. Nevertheless, assessing the soluble inorganic PHOS fraction of commercial diets requires specialized analytical methods that are not routinely performed by commercial laboratories. Although similar mechanisms may also apply to APHs, currently available evidence remains insufficient to support definitive conclusions.

The strong positive correlation observed between CREA and PHOS concentrations further supports the interpretation that hyperphosphatemia in affected hedgehogs was associated with impaired renal excretory function. Similar relationships between PHOS retention and declining renal function have been described in other species with CKD [31].

Calcium-to-phosphorus balance may also represent a relevant nutritional factor in APHs. In most vertebrates, including insectivorous species, recommended dietary calcium-to-phosphorus ratios generally range between 1:1 and 2:1 to support mineral homeostasis and prevent metabolic disturbances [9,34]. In the present study, the APH-specific diet had a higher calcium-to-phosphorus ratio than the commercial kitten diet. Nevertheless, serum calcium concentrations did not differ significantly between dietary groups, suggesting that alterations in PHOS metabolism were more pronounced than disturbances in systemic calcium homeostasis. However, species-specific calcium and PHOS requirements have not been established for APHs and are likely influenced by their omnivorous–insectivorous feeding ecology. In the wild, APHs consume a diverse diet consisting of invertebrates, small vertebrates, and plant material [3], suggesting that their mineral requirements may differ from generalized recommendations extrapolated from other taxa. Further nutritional studies are therefore needed to better define calcium and PHOS requirements in this species.

Differences in dietary fiber content and composition may also have contributed to the observed differences in renal biochemical markers between the two dietary groups. Commercial kitten diet typically contains variable content of plant-derived fibers primarily intended to support digestion and hairball management [35]. In contrast, due to the absence of a caecum, APHs are considered anatomically adapted to an insectivorous diet, with chitin serving as a physiologically relevant fiber source. However, limited utilization of cellulose has been suggested [36]. Dietary fiber has been recognized as a modulator of renal health through interactions with the gut microbiome and uremic toxin production in humans and companion animals [37,38,39]. Although fiber-related mechanisms were not directly evaluated in the present study, the substantially lower fiber content and substantially different fiber sources in the commercial kitten diet (plant-based fiber) than those reported in the APH-specific diet (a mix of plant-based and insect-derived fiber, including dried mealworms) may represent an additional factor contributing to the observed alterations in renal function parameters.

Early detection of renal dysfunction in APHs remains difficult in clinical practice. Owners rarely present hedgehogs for routine wellness examinations, and subtle clinical signs such as polyuria, polydipsia, or gradual weight loss are often overlooked due to the species’ nocturnal behavior and limited observable activity [40]. Consequently, animals are frequently examined at more advanced stages of renal dysfunction, limiting opportunities to evaluate early disease progression and preventive dietary interventions.

Overall, the findings of this study indicate an association between dietary regimen and alterations in renal function parameters in APHs. While causality cannot be established due to the observational nature of the study, the consistent elevation of renal biochemical markers in hedgehogs fed a commercial kitten diet supports the recommendation of species-specific diets in clinical practice.

## 5. Limitations

The primary limitation of this study is the relatively small sample size, which was constrained by the inclusion of privately owned pet hedgehogs with consistent long-term dietary histories. Given the small body size of APHs, blood sampling was intentionally limited to the volume required for serum biochemical analyses in order to minimize animal burden. As the primary objective of the study was the evaluation of renal biochemical markers, complete blood counts were not performed in all animals and were therefore not included in the analyses. Additionally, the lack of a prospective study design with longitudinal follow-up precludes assessment of disease progression and temporal relationships between diet and renal dysfunction. The duration of dietary exposure was owner-reported and could not be objectively standardized among individuals. Although dietary calcium and PHOS contents were available through manufacturer declarations and targeted laboratory analysis, comprehensive laboratory characterization of all nutritional components and mineral fractions in both diets was not performed. The absence of validated species-specific reference ranges and biomarkers for renal dysfunction in APHs further limits interpretation and necessitates partial extrapolation from human and companion animal nephrology literature. Nevertheless, feeding a commercial kitten diet remains a common practice in captive APHs and is still referenced in some husbandry recommendations. In this context, the present findings are considered clinically relevant, as they highlight a potential nutritional risk that warrants further investigation and increased awareness among veterinarians and owners. Because animals were client-owned and presented for either wellness evaluation or non-specific clinical concerns, residual confounding by disease severity at presentation cannot be excluded. As the study population consisted of client-owned hedgehogs presented to a referral hospital, the findings may not be fully representative of the general captive APH population. Future studies should include comprehensive nutritional characterization of commercial diets, with particular emphasis on fiber composition (plant-derived vs. chitin) and PHOS fractions (organic vs. inorganic, soluble), to better elucidate the relationship between dietary composition and CKD progression in APHs.

## 6. Conclusions

African pygmy hedgehogs fed a commercial kitten diet exhibited significantly higher serum concentrations of BUN, CREA, and PHOS compared with animals fed an APH-specific diet. These findings suggest that feeding commercial kitten diets may be associated with unfavorable renal biochemical profiles in APHs. Given the increasing popularity of this species as a companion animal and the frequent occurrence of renal pathology, dietary management should be considered an essential component of renal health maintenance in APHs. The observed differences in PHOS-related parameters occurred despite the absence of significant differences in serum calcium concentrations, further supporting altered PHOS handling as a potential feature of renal dysfunction in affected animals. Further controlled, prospective studies with larger sample sizes are needed to define species-specific nutritional requirements, establish validated renal reference ranges, and develop evidence-based guidelines for the prevention and management of renal disease in this species.

## Figures and Tables

**Table 1:** Nutritional composition of the commercial kitten diet and the African pygmy hedgehog-specific diet used in the study.

Analytical Constituents	Kitten Diet (%)	APH-Specific Diet (%)
Crude protein	36.00	41.10
Crude fat	14.00	10.00
Crude fiber	0.90	3.80
Crude ash	7.30	8.67 *
Calcium	1.25	2.64 *
Phosphorus	1.00	1.15 *

* Crude ash, calcium and phosphorus contents of the APH-specific diet were determined by targeted commercial laboratory analysis on native/as-fed samples. Phosphorus was analyzed using a spectrometric method according to SRPS ISO 6491:2002 [12]. Other nutritional values were obtained from manufacturer declarations.

**Table 2:** Clinical presentation, physical examination findings, and renal ultrasonographic abnormalities of African pygmy hedgehogs included in the study.

Diet Group	Presenting Clinical Findings	Renal Ultrasonographic Findings
Kitten diet	Hindlimb weakness, good body condition, adequate hydration	Irregular renal contours
	Hyporeactivity, hypothermia, palpable hepatomegaly and renomegaly	Irregular renal contours and bilateral renal cysts
	Oral mass, abdominal effusion	Reduced corticomedullary differentiation
	Lethargy, hypothermia, generalized pallor	Reduced corticomedullary differentiation
	Reduced appetite, weight loss	Altered renal parenchymal echogenicity
	Hindlimb locomotor deficits	Reduced corticomedullary differentiation
	Reduced appetite	Bilateral renal cysts
	Mild weight loss, dermatological lesions	Bilateral renal parenchymal alterations and a right renal cyst
	Good body condition, chronic ocular lesion	Renal cysts
	Diarrhea	Irregular renal contours
APH-specific diet	Weight loss, dehydration, muscle atrophy	Altered renal parenchymal echogenicity
	Palpable hypogastric mass, bilateral lingual abscesses	Altered renal parenchymal echogenicity
	Vomiting, weight loss	Renal cysts
	Abdominal mass, severe oral lesions	Altered renal parenchymal echogenicity
	Progressive weight loss	Reduced corticomedullary differentiation
	Reduced appetite, moderate dehydration, lethargy, positive urine dipstick findings (hematuria and proteinuria)	Atrophic right kidney with loss of corticomedullary differentiation, mild increased echogenicity of the left kidney
	Tremor, low posture, absent proprioception	Altered renal parenchymal echogenicity
	Reduced appetite, weight loss	Reduced corticomedullary differentiation
	Oral mass	Altered renal parenchymal echogenicity

Clinical findings include the presenting complaints and physical examination findings recorded at admission. Renal ultrasonographic findings were evaluated qualitatively, as validated species-specific reference values for renal size, parenchymal echogenicity, and Doppler parameters have not yet been established for APHs.

## Data Availability

The data presented in this study are available within the article. Additional information is available from the corresponding author upon reasonable request.
